# Microparticles Expressing IL-1β Are Linked to Neuroinflammation and Cognitive Decline After Carbon Monoxide Poisoning

**DOI:** 10.3390/ijms27177880

**Published:** 2026-09-03

**Authors:** Awadhesh K. Arya, Kinjal Sethuraman, Jaylyn Waddell, Abid R. Bhat, Zuha Imtiyaz, Deepa Walia, Yuanyuan Liang, Yong Sung Cha, Yoonsuk Lee, Siamak Moayedi, Douglas Sward, Stephen R. Thom

**Affiliations:** 1 Department of Emergency Medicine, University of Maryland School of Medicine, Baltimore, MD 21201, USA; aarya@som.umaryland.edu (A.K.A.); ksethuraman@som.umaryland.edu (K.S.); jwaddell@som.umaryland.edu (J.W.); arbhat@som.umaryland.edu (A.R.B.); zimtiyaz@som.umaryland.edu (Z.I.); dwalia@som.umaryland.edu (D.W.); mak.moayedi@som.umaryland.edu (S.M.); dsward@som.umaryland.edu (D.S.); 2 Department of Epidemiology and Public Health, University of Maryland School of Medicine, Baltimore, MD 21201, USA; yliang@som.umaryland.edu; 3 Department of Emergency Medicine and Research, Institute of Hyperbaric Medicine and Science, Yonsei University Wonju College of Medicine, Wonju 26426, Republic of Korea; emyscha@yonsei.ac.kr (Y.S.C.); yslee524@gmail.com (Y.L.)

**Keywords:** neurodegeneration, extracellular vesicles, cytokine, microparticles, neutrophils, neurological sequelae

## Abstract

Immune mediators are elevated due to carbon monoxide (CO) poisoning but their role in the development of neurological deficits is unknown. Circulating microparticles (MPs) are elevated in response to CO and correlate with the development of neurological complications. We hypothesized that interleukin (IL)-1β is carried by MPs and contributes to the development of neurological sequelae (NS). Blood-borne IL-1β in CO-poisoned patients is almost exclusively carried by MPs. Healthy humans have 0.8 ± 0.8 (*n* = 27) MPs/μL bearing IL-1β on the membrane surface. IL-1β-bearing MPs obtained from patients at the time of CO poisoning diagnosis who recover (Global Deterioration Score [GDS] = 1 at 1 month) numbered 35 ± 12/μL (*n* = 27, *p* < 0.001 vs. control) and patients who sustained NS (GDS > 1 at 1 month) had 75 ± 38/μL (*n* = 27; *p* < 0.001 vs. control and CO-recovered groups). Twice as many MPs carry IL-1β cargo as express surface-bearing IL-1β. The concentration is higher post-CO but does not correlate with NS. Those with NS have 43.8 ± 42.0 pg IL-1β /10^6^ MPs, and those with GDS = 1 have 33.0 ± 32.0 pg/10^6^ MPs, versus controls 5.4 ± 0.3 pg/10^6^ MPs, *p* < 0.001. In a murine CO model, MPs IL-1β cargo follows similar proportions to humans. Pharmacologic blockade of IL-1β using an IL-1 receptor antagonist or neutralizing anti-IL-1β antibody inhibited neuroinflammation and preserved neurological function. Results demonstrate that brain-derived MPs liberated via the glymphatic system to the peripheral circulation activate neutrophils which are the prime generators of IL-1β-bearing MPs that maintain a cycle of neuroinflammation. Targeting IL-1β disrupts MP-mediated inflammation.

## 1. Introduction

Carbon monoxide (CO) is among the most prevalent poisons worldwide, affecting ~160/100,000 people annually, with a somewhat higher incidence in some developing countries [1,2,3,4,5]. Intentional poisonings account for the majority of deaths, which often are due to cardiac insults [1,6]. Accidental exposures represent the majority of non-fatal poisonings [7], and cost over $1 billion annually in the United States for direct hospital care and lost earnings [8]. Morbidity is related to neurological injuries [9,10]. At high concentrations, CO causes mitochondrial dysfunction leading to direct cytotoxicity; however, neurotoxicity typically involves oxidative stress due to reactive species from mitochondria and NADPH oxidase, and under special circumstances xanthine oxidase, leading to inflammatory responses [11,12,13,14,15,16,17,18,19,20,21,22]. Interleukin (IL)-1β, IL-6, TNFα and NF-κB are elevated in the cerebral cortex and hippocampus in animal models [23,24,25]. Patients with encephalopathy exhibit increases in IL-1β, IL-6, IL-10 and IL-13 in cerebrospinal fluid (CSF) and/or plasma [26,27,28].

A causal relationship between CO poisoning and inflammatory cytokines has not been demonstrated, but a recent bidirectional two-sample Mendelian randomization assessment involving 231 CO cases implicated IL-1β, IL-10 and IL-13 as playing roles in CO poisoning prognosis [29]. Among these three, IL-1β stands out as having only pro-inflammatory activity. IL-1β promotes leukocyte activation, microglial priming, endothelial dysfunction, and synaptic impairment following ischemic, traumatic, and toxic insults [30,31]. Importantly, mature IL-1β is generated through activation of the nucleotide-binding oligomerization domain-like receptor, pyrin domain containing (NLRP) 3 inflammasome, a multiprotein complex that mediates caspase-1-dependent cleavage of pro-IL-1β into its bioactive form [32,33,34]. Unlike most secreted cytokines, IL-1β lacks a conventional signal peptide, precluding its release through the classical endoplasmic reticulum–Golgi secretory pathway [32,35,36,37]. Consequently, IL-1β relies on noncanonical secretion mechanisms with export via extracellular vesicles such as microparticles (MPs).

In a study of 114 CO patients, the number of blood-borne MPs expressing myelin basic protein (MBP) on the membrane surface was shown to correlate with the development of neurological morbidity [38]. Further, MPs appear to play a causal role in neuroinflammation and functional neurological deficits in a murine CO poisoning model. Results in the murine model indicate that the initial CO insult stimulates MPs production in the brain [38,39,40,41,42]. The MPs pass from central nervous system (CNS) glymphatic channels to the deep cervical lymph nodes (DCLNs) and then to the systemic circulation. Brain-derived, thrombospondin-1-expressing MPs cause neutrophil activation with production of additional MPs, some expressing filamentous (F)-actin. The F-actin MPs lead to neutrophil autoactivation, exacerbating the inflammatory process. Activated neutrophils and F-actin MPs adhere to the brain microvasculature endothelium to disrupt the blood–brain barrier (BBB). Concurrent release of F-actin and MBP-expressing MPs from the brain activate peripheral lymphocytes that subsequently gain CNS access through the ruptured BBB to contribute to on-going neuroinflammation lasting weeks that causes functional compromise [38,41].

With these issues as a background, we hypothesized that IL-1β plays a role in CO-induced neuroinflammation. Therefore, we sought evidence on whether MPs-associated IL-1β is linked to neurological outcome. We still had 27 blood samples from a recent study where we found that the number of F-actin and MBP-expressing MPs was correlated with 1-month neurological outcome following CO poisoning [38]. We used these samples and found a relationship between IL-1β and outcome, so went on to investigate the relationship between IL-1β, neuroinflammation and functional outcome in a murine CO poisoning model and whether IL-1β inhibition modifies the response to CO exposure.

## 2. Results

### 2.1. Circulating IL-1β in CO Patients

We retrospectively evaluated IL-1β using blood from 27 CO-poisoned patients who sustained neurological sequelae (NS) within 1 month after exposure. These were blood samples obtained in the Emergency Department (ED) at time of diagnosis and NS was determined based on neurological symptoms and having a Global Deterioration Score (GDS) between 2 and 7 (see Materials and Methods section). Values were compared against 27 age- and sex-matched CO patients obtained at a similar time as those with NS but who recovered (GDS = 1) at 1 month and 27 similarly matched controls. Table 1 lists assessments of clinical status and laboratory measurements for the CO groups (GDS = 1 and GDS > 1). Note this is a subset of information on CO patients from the earlier study [38]. The only statistically significant difference between these groups was the troponin value, which was nearly 10-fold higher in those who sustained NS. Supplemental Table S1 lists clinical results for all CO patients, demonstrating no significant differences in the two groups with IL-1β measurements in the table below from the larger groups in the earlier report. Supplemental Table S2 shows the distribution of GDSs for the 27 with poor outcome in the table below versus the larger group (*n* = 37) in our earlier report.

**Figure 1:**
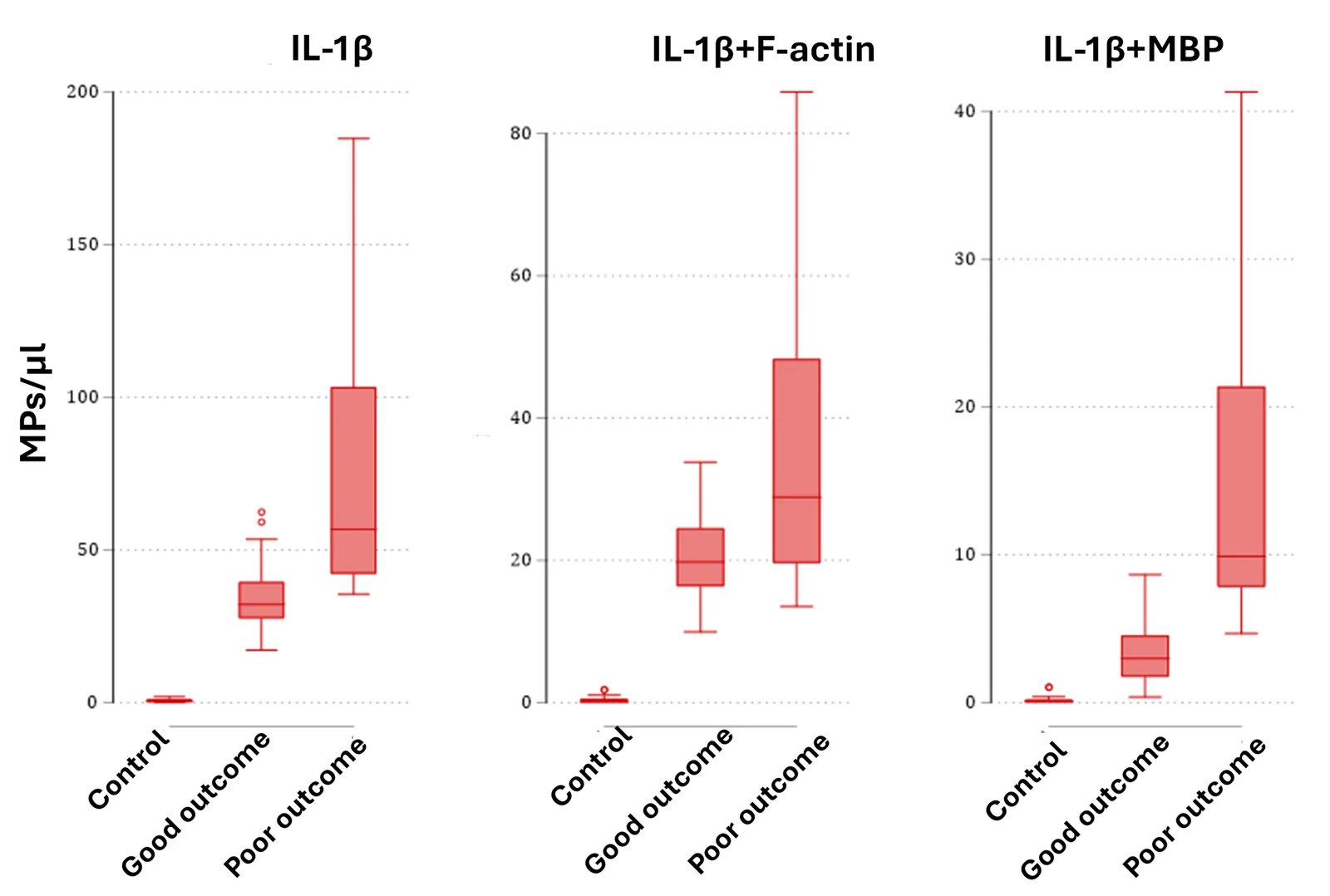
Boxplots of IL-1β-MP values of admission blood samples from three groups (*n* = 27 each group): healthy controls vs. CO without NS by 1-month post-exposure (CO good) vs. CO with NS by 1-month post-exposure (CO worse). Each box represents the interquartile range (IQR 25th–75th percentile), with median indicated by line inside the box. Whiskers extend to the most extreme data points within 1.5 X IQR from the box, and values outside this range are plotted as individual circles (outliers). Median values of IL-1β-expressing MPs for all three groups were significantly different (*p* < 0.001, Kruskal–Wallis H test) and pairwise comparisons were also significantly different after Bonferroni adjustment (healthy control vs. CO good: *p* < 0.001; healthy control vs. CO worse: *p* < 0.001; CO good vs. CO worse: *p* = 0.0013). Values for subgroups expressing IL-1β and myelin basic protein (MBP) were also significantly different for each group (*p* < 0.001), and IL-1β plus F-actin significance was as follows: healthy control vs. CO good: *p* < 0.001; healthy control vs. CO worse: *p* < 0.001; CO good vs. CO worse: *p* = 0.0502.

Consistent with reliance on its noncanonical secretion mechanism, IL-1β in blood samples appears to be carried by MPs. There was no detectable IL-1β in supernatants after MPs removal from plasma (see Materials and Methods section). The concentration of IL-1β was significantly higher in MPs from CO patients (Table 1) versus controls, 5.4 ± 0.3 pg/10^6^ MPs (*n* = 27; *p* < 0.001). However, the levels were not statistically significantly different between the two CO groups. This relationship differed when examining IL-1β expressed on the surface rather than the total present in lysed MPs. Figure 1 exhibits distributions of blood-borne MPs expressing IL-1β on the membrane surface for controls and CO patients. Pairwise comparison (Dunn’s method) with Bonferroni adjustment demonstrated that there were significantly different values for IL-1β-expressing MPs in each group (healthy control vs. CO good: *p* < 0.001; healthy control vs. CO worse: *p* < 0.001; CO good vs. CO worse: *p* = 0.0013). For MPs expressing IL-1β and F-actin or MBP, most groups were also significantly different from the control (see Figure 1). The sample size was insufficient to establish a relationship between IL-1β-expressing MPs and clinical parameters such as troponin, intubation, loss of consciousness, and GCS.

To assess the proportions of MPs expressing surface IL-1β versus intra-particle cytokine, MP samples from five healthy controls and five NS-sustaining CO patients were assayed by flow cytometry with one fraction undergoing a permeabilization step to assess total versus surface IL-1β. Surface IL-1β was present on 0.06 ± 0.04% of control MPs versus 5.8 ± 0.3% (*p* = 0.008) of MPs from CO patients. Permeabilized control MPs had detectable IL-1β in 0.4 ± 0.1% of particles while 10.4 ± 1.0% (*p* < 0.001) of CO MPs had detectable IL-1β. Because the total number of MPs in CO patients was nearly three times higher than in controls (569 ± 36/μL plasma in controls, 1491 ± 89/μL plasma in CO cases), the fractions of IL-1β-bearing MPs were starkly magnified: 2.5 ± 0.6 MPs/μL plasma in controls, 156 ± 17 MPs/μL plasma in CO cases (*p* < 0.001).

Prior work has demonstrated neutrophil activation occurs in CO patients, but no statistical difference was identified between those with NS versus patients who recovered [41]. However, because of data showing the pathological role for neutrophil activation [38,41], we investigated whether IL-1β cargo differed between MPs expressing the neutrophil protein CD66b from those that do not. CD66b-expressing MPs were assayed from nine CO patients (four NS-sustaining, five GDS = 1). CD66b-negative MPs carried undetectable levels of IL-1β (detection threshold 1.2 pg/mL), whereas the concentration in the CD66b-positive MPs was 82.2 ± 9.4 pg/million MPs. There was no significant difference between NS-sustaining CO patients and those with a good outcome.

### 2.2. Murine CO Model, MPs and IL-1β Antagonists

**Figure 2:**
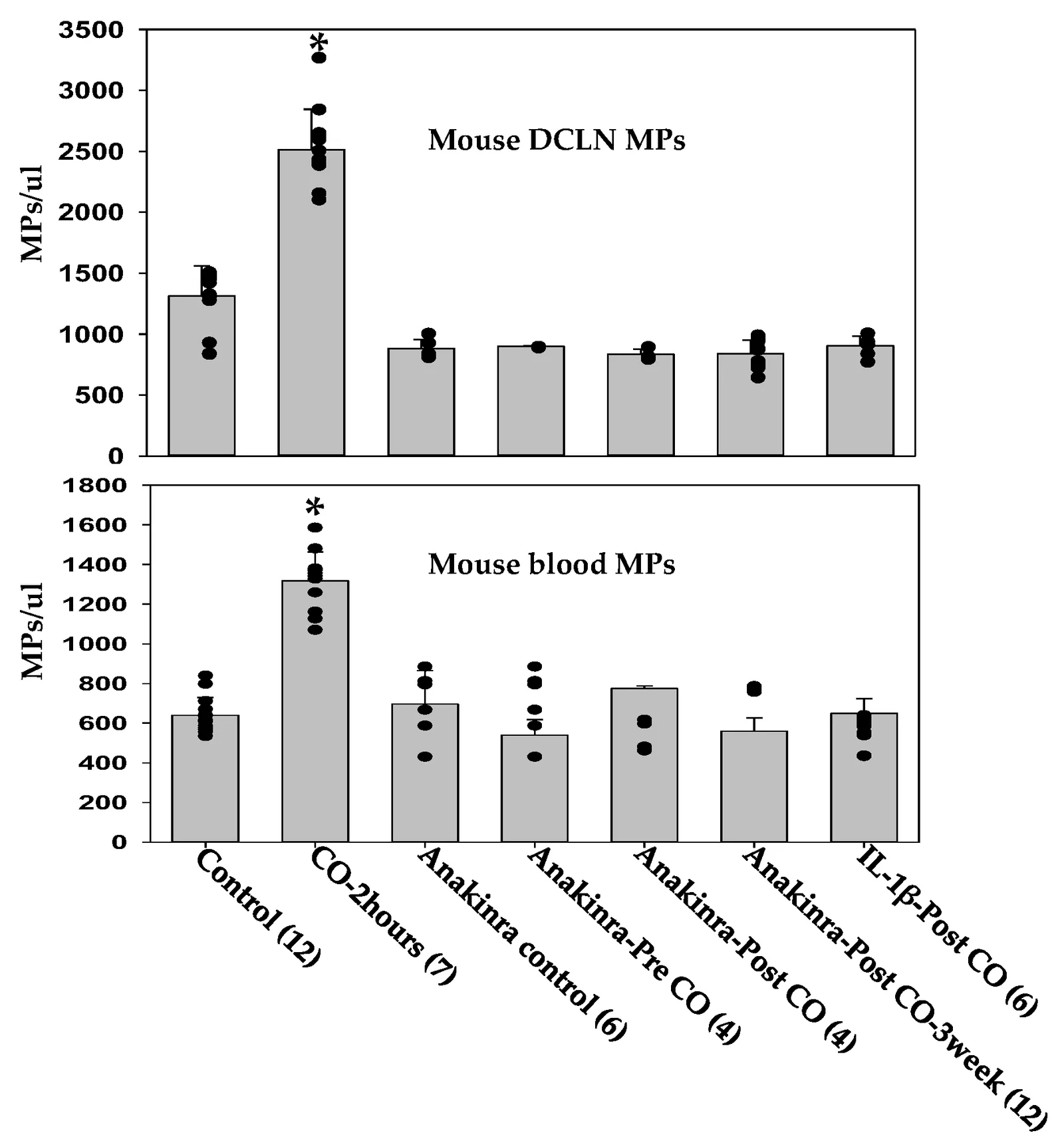
Deep cervical lymph node (DCLN) and blood microparticles (MPs). The total numbers of MPs in deep cervical nodes (DCLNs) and blood are shown. The data represent mean ± SD (*n* for each group shown along the abscissa), where * *p* < 0.01 vs. control (Kruskal–Wallis test). Subgroups of MPs expressing protein markers are shown in Table 1 (A and B). Groups include wild-type control (control); mice exposed to CO and euthanized 2 h later (CO-2hours), wild-type mice injected with a sterile solution of anakinra (100 mg/kg) intraperitoneal (Anakinra control), mice injected with a sterile solution of anakinra and then exposed to CO (Anakinra-Pre CO) or mice exposed to CO and immediately injected with a sterile solution of anakinra (Anakinra-Post CO) and euthanized 2 h later; mice exposed to CO, injected with anakinra immediately after the CO exposure, and euthanized 3 weeks later (Anakinra-Post CO-3week); and mice exposed to CO, injected with anti-IL-1β antibody (50 μg/mice) immediately after the CO exposure and euthanized 2 h later (IL-1β-Post CO).

We next investigated the role of IL-1β in the murine CO model. MPs production was examined after exposure to CO as described in Methods. Increases in the number of circulating MPs in the deep cervical lymph node (DCLN) and blood at 2 h post-exposure are shown in Figure 2, with the distribution of surface-expressed proteins specific to alternative cell types displayed in Table 2 and Table 3. The concentration of IL-1β was 9.2 ± 3.6 pg/million MPs in CO-exposed mice, and it was undetectable in MPs from control mice.

Due to the clinical observations pertaining to surface-expressed IL-1β on MPs, we evaluated surface versus total IL-1β expression by Ly6G (the murine equivalent to CD66b) -expressing MPs from five control and five CO-exposed mice. IL-1 β was identified on the surface of only 0.07 ± 0.24% of control Ly6G-positive MPs and 0.69 ± 0.24% of permeabilized particles. Surface IL-1β was identified on 4.1 ± 1.1% (*p* = 0.008) of CO-exposed Ly6G-positive MPs and 8.8 ± 1.9% of permeabilized MPs (*p* = 0.008). There were slightly more than double the total number of MPs in CO-exposed mice than in controls (694 ± 75/μL plasma in controls, 1405 ± 101/μL plasma in CO mice), which magnified the fractions of IL-1β-bearing MPs. Surface-expressing particles numbered 0.4 ± 0.7 MPs/μL plasma in controls, and 56 ± 11 MPs/ μL (*p* < 0.001) plasma in CO mice; and total IL-1β-bearing MPs numbered 4.9 ± 1.9/ μL plasma in control mice and 124 ± 28/ μL (*p* = 0.008) plasma in CO-exposed mice. Ly6G-negative MPs from CO-exposed mice carried surface IL-1β on just 0.11 ± 0.07% of particles and 0.31 ± 0.07% in permeabilized particles. None was detected in or on Ly6G-negative MPs from control mice.

The role of IL-1β in CO-induced inflammation was assessed by evaluating the impact of intraperitoneal injections of anakinra, an IL-1 receptor antagonist, administered either pre- or post-CO exposure and the effect of neutralizing antibody to IL-1β administered post-CO exposure. As shown in Figure 2, Table 2 and Table 3 these interventions attenuated CO-induced MPs generation in both blood and DCLN.

Because intra-CNS MPs drive systemic inflammation once liberated via the glymphatic system, we also evaluated IL-1β presence in MPs from DCLN [38,39,40,41,42]. IL-1β was not detectable in F-actin-negative MPs in CO-exposed mice nor in any MPs from control mice (*n* = 5). The concentration of IL-1β F-actin expressing DCLN MPs from CO-exposed mice was 1/5th that of peripheral blood, 1.8 ± 1.6 pg/million MPs.

### 2.3. Murine Neutrophil Activation and IL-1β Antagonists

**Figure 3:**
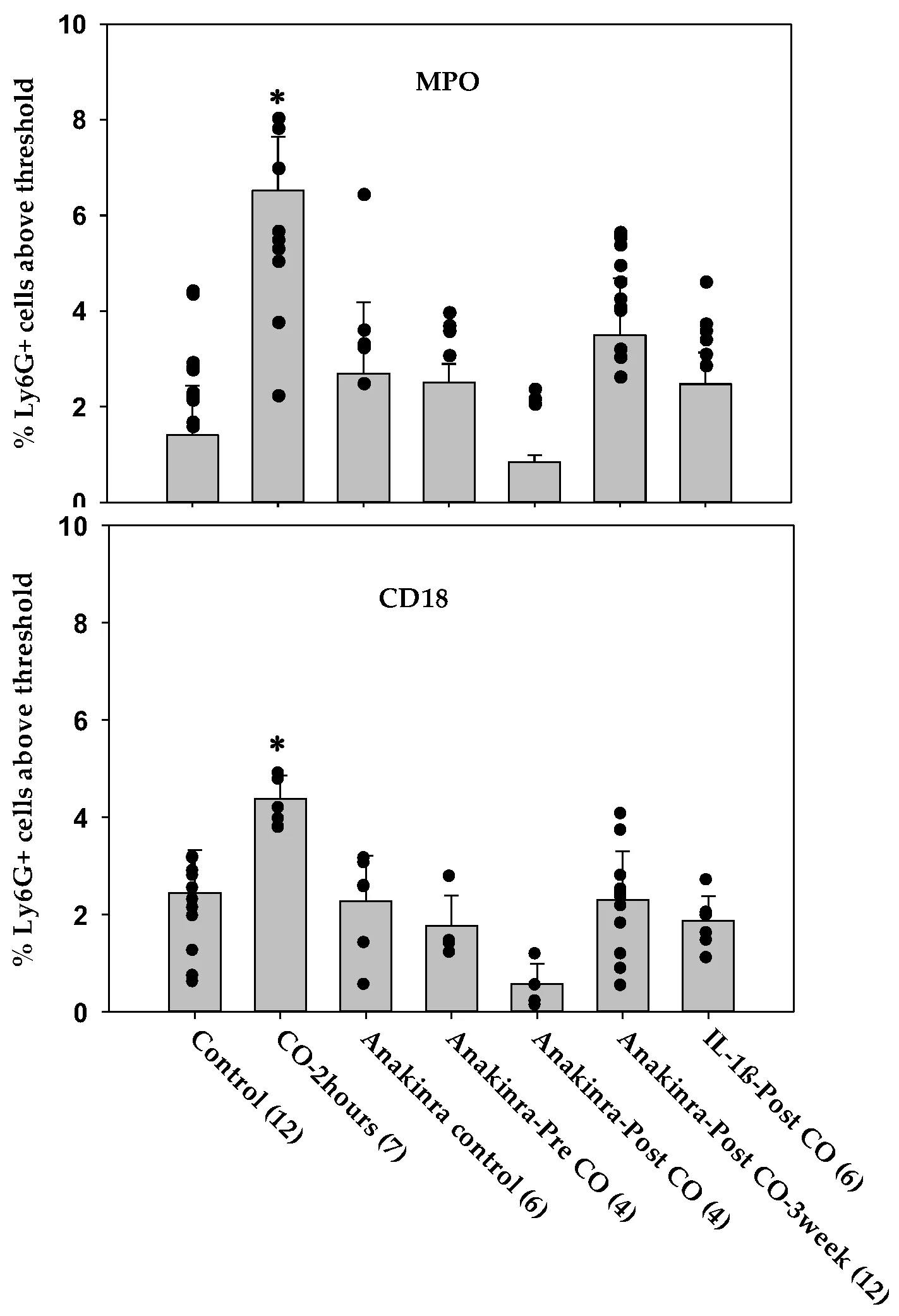
Neutrophil activation: Neutrophil activation was assessed by flow cytometry as the % of Ly6G-positive cells expressing myeloperoxidase (MPO) and CD18 on the membrane surface above a threshold concentration present on control cells. Mouse groups and data are as described in Figure 1. * *p* < 0.01 vs. control (Kruskal–Wallis test).

Neutrophil activation in mice was assessed by flow cytometry as an increase in surface expression of CD18 (a component of β_2_ integrins), and myeloperoxidase (MPO), indicative of cell degranulation. Significantly higher percentages of CD18 and MPO on the neutrophil surface were observed in CO-exposed mice (Figure 3) as compared to the control. Expression of the neutrophil activation markers was significantly reduced in mice exposed to CO when treated with the anakinra or anti-IL-1β antibody.

### 2.4. Assessment of Vascular Injury

**Figure 4:**
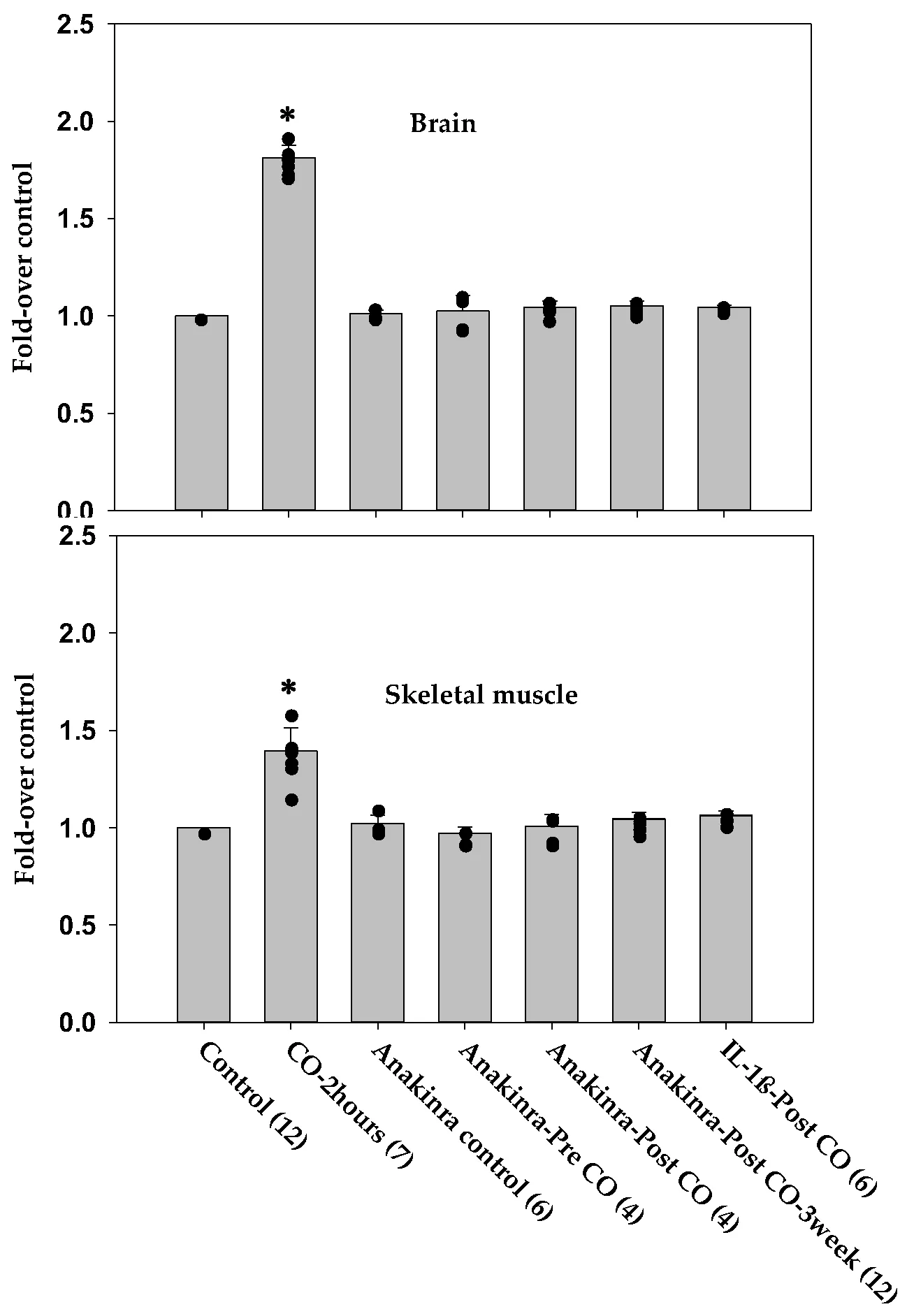
Capillary leakage in mice brain and skeletal muscle. Extravasation of 2 × 10^6^ Da rhodamine-labeled dextran in brain and leg skeletal muscle was evaluated as described in Materials and Methods from mouse groups as described in Figure 1. Values reflect the fold difference in rhodamine-dextran/mg tissue protein (mean ± SD) versus the values in control mice processed concurrently with each experimental group. * *p* < 0.01 vs. control (Kruskal–Wallis test).

A statistically significant increase (*p* < 0.01) in vascular leakage was observed in CO-exposed mice compared with controls, consistent with prior findings [41,42]. Mice receiving either anti-IL-1β antibody or anakinra showed no detectable vascular leakage at 2 h post-CO exposure, similar to controls (Figure 4). The protective effect of anakinra persisted for up to three weeks after CO exposure, indicating sustained vascular stabilization (Figure 4).

### 2.5. Lymphocyte Proliferation

**Figure 5:**
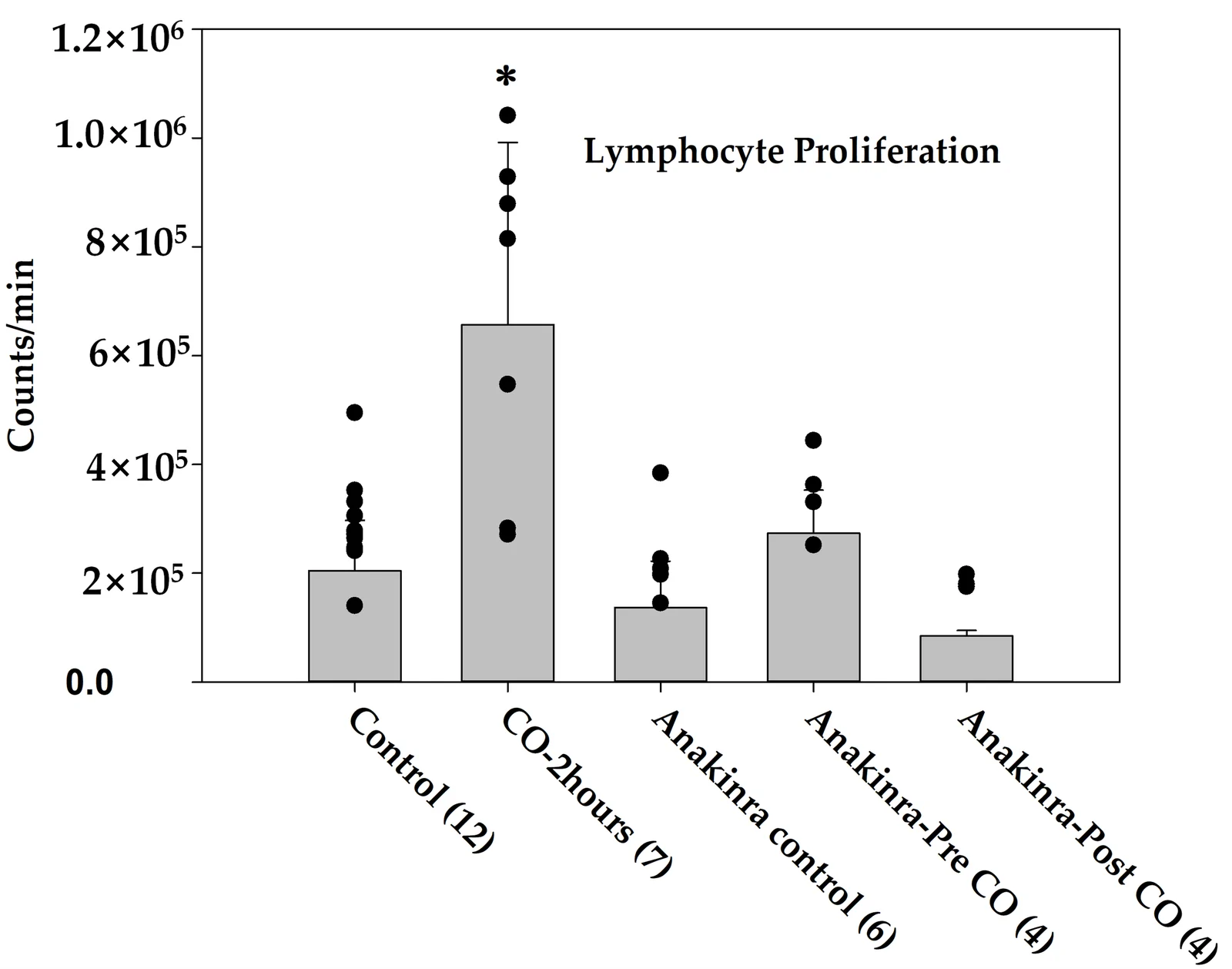
Lymphocyte proliferation. Lymphocyte proliferation in the cells obtained from lymph nodes were assessed by beta scintillation counter following methods mentioned in the Materials and Methods section. Mouse groups and data are as described in Figure 1. * *p* < 0.01 vs. control (Kruskal–Wallis test).

To assess whether IL-1β contributed to CO-induced adaptive immune activation in response to MBP, proliferation of lymphocytes isolated from peripheral lymph nodes was assessed as [^3^H]-thymidine incorporation challenged with bovine MBP versus ovalbumin (control). Lymphocytes from CO-exposed mice exhibited a marked increase in proliferative activity, which was inhibited by anakinra (Figure 5).

### 2.6. CO-Induced Neuroinflammation

**Figure 6:**
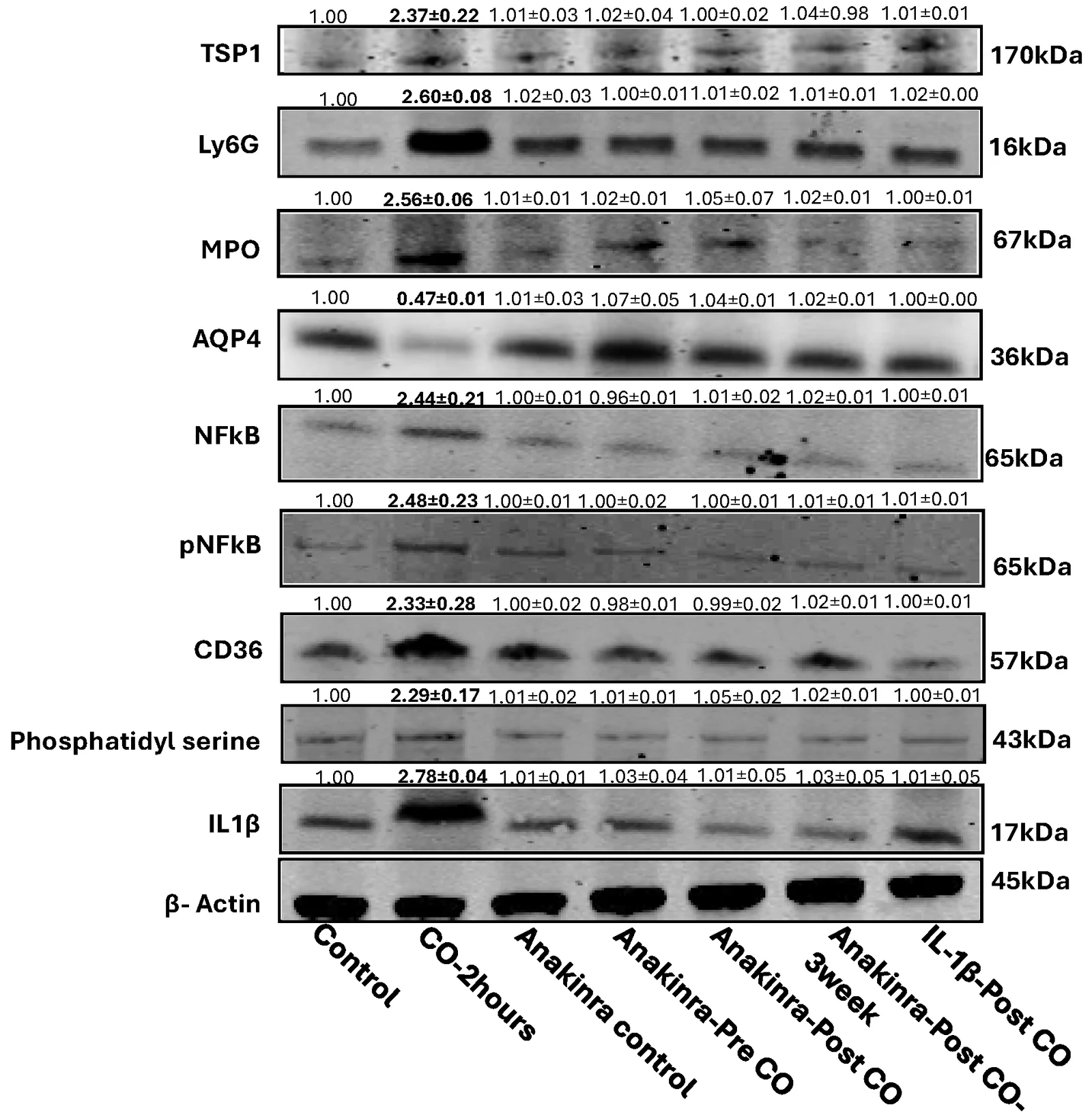
Western blots of brain homogenates to assess neuroinflammation. This is a representative Western blot of brain homogenates for mouse groups as described in Figure 1 with mean ± SD values shown above each band expressed as fold-change from control for replicate studies. Normalization of band densities to β actin in the same samples was performed for daily experiments and then values for experimental groups were expressed as a ratio against the control value (thus control is shown as 1.0). Bold numbers indicate values statistically significantly different from control (*p* ≤ 0.01, Kruskal–Wallis test). Blots were probed for thrombospondin-1 (TSP1), Ly6G, myeloperoxidase (MPO), Aquaporin-4 (AQP4), NF-κB, p65-phosphorylated NF-κB (pNF-κB), CD36, phosphatidyl serine, and IL-1β proteins.

**Figure 7:**
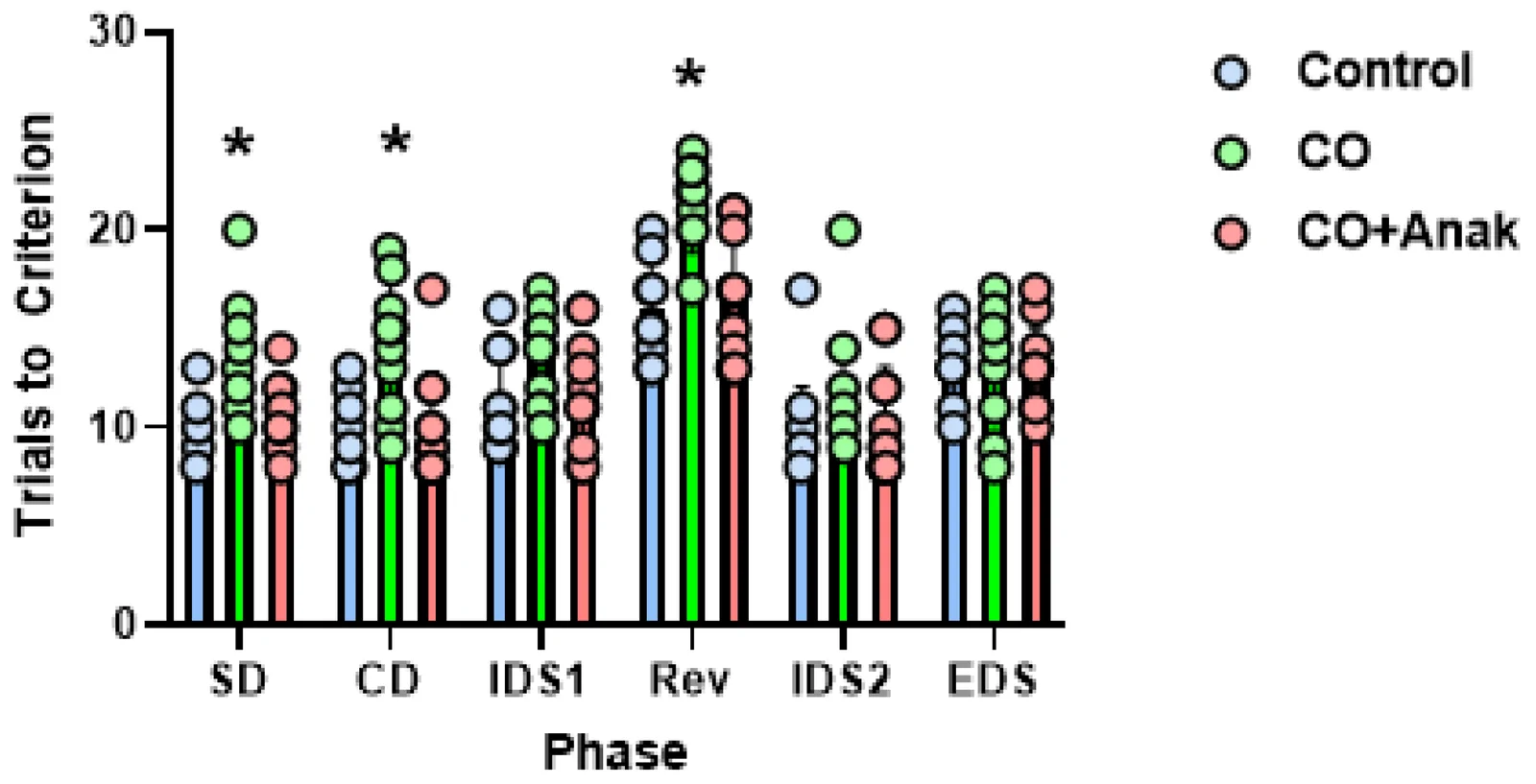
Attentional set shift scores. CO exposure significantly impaired performance on the Attentional Set Shifting and Reversal Task and this impairment was reversed by intraperitoneal injection of anakinra (100 mg/kg). Mice progressed through a series of discriminations motivated by a food reward. The number of trials to reach a criterion of 8 correct responses in a row were recorded. CO-exposed mice were significantly different from control (Tukey’s post hoc comparison, *p* < 0.01). Analysis of individual phases confirmed that CO-exposed mice differed from non-exposed mice on the Simple Discrimination (SD), Compound Discrimination (CD) and reversal phase of the task; Tukey’s post hoc comparison, * = *p* < 0.001. Bars show the mean with individual data points for each test. Anakinra-treated mice were not different from non-exposed control mice.

Consistent with our prior work [41,42], Western blot analyses of endothelium-enriched brain homogenates from CO-exposed mice revealed significant upregulation of key inflammatory and vascular injury markers including thrombospondin-1 (TSP-1), total NF-κB, phosphorylated p65 NF-κB, thrombospondin 1 (TSP1), and the scavenger receptor CD36 (Figure 6). These changes were accompanied by a marked loss of aquaporin-4 (AQP4), indicating astrocytic end foot disruption, and evidence of neutrophil sequestration demonstrated by elevations in MPO and Ly6G expression (Figure 7). In addition, CO-exposed mice exhibited significantly higher levels of phosphatidylserine (PS), an index of MP accumulation, and IL-1β. All of these neuroinflammatory changes were abated in mice treated with anakinra or anti-IL-1β antibody (Figure 6). Notably, the neuroprotective effects of IL-1β receptor blockade were not transient; anakinra treatment preserved suppression of neuroinflammatory markers in mice three weeks post-CO (Figure 6).

### 2.7. Cognitive Deficits Following CO Exposure

Executive function impairment is a well-recognized and frequently reported complication among survivors of CO poisoning [43,44,45]. To evaluate comparable cognitive domains in mice, we employed the Attentional Set Shifting and Reversal Task (ASSTR), a validated paradigm for assessing problem solving ability and cognitive flexibility [46]. Performance scores for each phase of the ASSTR are presented in Figure 7. For statistical analyses, air-exposed controls were combined with air-exposed mice treated with anakinra, as anakinra treatment alone did not alter performance. Final group sizes were control, *n* = 13; CO-exposed, *n* = 12; CO + anakinra, *n* = 11.

Repeated measures ANOVA across the six ASSTR phases revealed a main effect of phase, *F*(5,165) = 50.69, *p* < 0.001, confirming changes in the number of trials required to reach the criterion, reflecting differences in task difficulty. A significant phase × treatment interaction was also observed, *F*(10,165) = 2.12, *p* < 0.026, indicating that the pattern of performance across phases differed by experimental condition. The main effect of treatment was significant, *F*(2,33) = 15.8, *p* < 0.001.

Tukey’s post hoc comparisons of overall task performance (collapsed across phases) showed that CO-exposed mice required significantly more trials to reach criterion than controls (*p* < 0.001). Importantly, mice treated with anakinra following CO exposure performed significantly better than untreated CO-exposed mice (*p* < 0.001) and did not differ from control mice (*p* < 0.8), indicating substantial cognitive protection. Analysis of each phase separately revealed significant treatment effects during the simple discrimination (*F*(2,33) = 10.65, *p* < 0.001), compound discrimination (*F*(2,33) = 9.46, *p* < 0.001), and reversal learning (*F*(2,33) = 14.3, *p* < 0.001) phases. These phases are likely more challenging due to the novelty of stimuli and changes in contingencies. Reversal learning is particularly sensitive to prefrontal cortex dysfunction [47]. Taken together, these results demonstrate that CO exposure induces marked deficits in executive function-related behaviors in mice, and that IL-1β receptor blockade with anakinra effectively prevents these CO-induced cognitive impairments.

## 3. Discussion

This study identifies a previously unrecognized mechanism linking CO exposure to sustained neuroinflammation and cognitive impairment through the generation of IL-1β-expressing MPs. The murine findings indicate that targeting IL-1β may have benefit for diminishing CO-mediated NS. This extends our prior work suggesting that therapeutic use of recombinant human plasma gelsolin to lyse the neuroinflammatory MPs may be helpful [41]. The expression of IL-1β on MPs may aid with diagnosis of serious CO poisoning, as does MBP; however, the sample size in our study was insufficient to assess the role of clinical parameters such as loss or consciousness, or troponin levels in the outcome. Therefore, the results must be viewed as exploratory.

On a more fundamental immunological level, two findings stand out from our results. The first is that neutrophils appear to be the primary source of IL-1β in response to CO. This assessment is based on finding that IL-1β was only detected with MPs and MPs expressing neutrophil protein markers (CD66b in humans, Ly6G in mice) are the predominant carriers. However, the data do not preclude additional cell sources because surface protein rearrangements occur among circulating MPs. The second, of course, is that it is surface-bound IL-1β and not total cargo that is correlated with the development of NS. There is no unified mechanism for IL-1β secretion despite data indicating that non-canonical export via extracellular vesicles is necessary [32,35,36,37]. This is largely because data indicate that IL-1β secreted into the circulation appears to be directly available, thus suggesting free access to bind to its receptor [35,48]. Our data showing that some IL-1β is exported on an MP’s surface and not sequestered behind a cell membrane satisfies experimental observations. This finding also provides understanding for the anti-inflammatory efficacy of anakinra and antibody directed to IL-1β. That is, their efficacy can be envisioned given that some IL-1β on the MPs surface are not physically separated from the IL-1 receptor.

That MPs are the principal IL-1β carrier may be a peculiarity for CO poisoning. Others have shown that macrophages respond to extracellular ATP and epithelial cells respond to sepsis by secreting IL-1β via exosomes [49,50] which are ~1/10th the size of MPs and derived from multivesicular bodies, whereas MPs are generated by evaginations of the plasma membrane caused by the flipping of the membrane lipid PS [35]. Our method of MPs separation leaves exosomes in the supernatant with soluble proteins, so exosomes are not a major IL-1β carrier in response to CO [51].

The antagonistic actions of anakinra and anti-IL-1β antibody suggest that numerous inflammatory actions of IL-1β play roles in CO-mediated NS. The initial CO insult stimulates MPs production in the brain [38,39,40,41,42]. The murine data show that there are scant MPs with IL-1β that pass from the CNS glymphatic channels to the DCLN. However, once the brain-derived, thrombospondin-1-expressing MPs cause neutrophil activation, the resulting neutrophil-derived MPs with IL-1β contribute to on-going neutrophil activation, endothelial dysfunction with diffuse capillary leak and BBB disruption. The IL-1β may also contribute to microglial priming and possibly synaptic impairment based on data showing CO-MPs will cause functional neurological impairments [38,39,40,41,42]. Abrogation of CO-exposed murine lymphocyte sensitization to MBP by anakinra indicates a role for IL-1β and is consistent with the cyclical nature of the neuroinflammatory process.

Our data also show that these events translate into impaired neurological function. CO-exposed mice displayed significant impairments in executive function, cognitive flexibility, and reversal learning domains that are highly dependent on prefrontal cortical integrity. These are the same functions frequently impaired in human CO poisoning survivors.

Limitations to our study include its small sample size and its retrospective analysis. A larger prospective study is planned in which we will assess the diagnostic accuracy of circulating MPs expressing IL-1β, F-actin and MBP alone and in combinations. Finally, we should mention that how IL-1β is expressed on the membrane surface of MPs will require substantial additional investigation. Its presence suggests a novel protein trafficking and systemic signaling process not previously appreciated for MPs production. There are seven amino acid residues essential for IL-1β binding to the IL-1 receptor that are clustered into a ‘T-shaped’ region [52]. It is likely that this portion of IL-1β is exposed when incorporated into the MPs membrane. Five amino acids are non-essential for binding, forming a perimeter around the binding region (Glu-51, Asn-53, Leu-110, Met-148, and Phe-150) [52]. Much has been written about the purely hydrophobic core of IL-1β that harbors water molecules important for its structure and function [53,54]. It may be important to examine hydrophobic exterior sites involved with stabilizing the protein within the cell membrane.

## 4. Materials and Methods

### 4.1. Materials

The majority of chemicals and their sources used in this study were listed in the previous publication [41]. In addition to those, we used anti-CD36 PE/Cyanine7 (Biolegend, San Diego, CA, USA, catalog no. 102616) and IL-1 β AF647 (Invitrogen, Waltham, MA, USA, catalog no. 51701842) antibody for flow cytometry, anti-phosphatidylserine (PS) antibody (1:250, CAU30389, Biomatik, Kitchener, ON, Canada), anti-IL-1 β antibody (1:1000, ab9722, Abcam, Waltham, MA, USA) for Western blot, mouse- and human-specific IL-1 β ELISA Kits (BMS6002, BMS224-2, Thermo Fisher Scientific, Waltham, MA, USA), Streck cell preservative (31280312, Streck, Inc., La Vista, NE, USA). Anakinra were purchased from Amgen (Thousand Oaks, CA, USA), and neutralizing anti-mouse IL- β antibody (BE-0246) and nonspecific control IgG (BE-0089) were purchased from BioXcell, Brandord, CT, USA.

### 4.2. Patients

Patients involved in this study were a subset of those described in a prior publication. Among 35 patients who had sustained NS described in the previous report, blood samples were still available for 27 that formed the basis for the clinical portion of this report. Informed consent from all patients was obtained after the nature and possible consequences of the studies were explained.

Patient Study: Acute CO poisoning was diagnosed according to patient history and elevated carboxyhemoglobin (COHb) level. Patient management was performed independently of the blood sample collections. Once CO poisoning was considered, all patients received O_2_ by tight-fitting non-rebreather face mask. They received at least one treatment with hyperbaric oxygen (HBO_2_) as soon as feasible if they had experienced loss of consciousness, manifested abnormal neurological signs or cognitive dysfunction, cardiovascular dysfunction, elevated cardiac enzymes, ischemic electrocardiogram changes, or had a COHb ≥ 25%. HBO_2_ treatments were performed at either 2.8 or 3.0 atmospheres absolute (ATA) for 60 to 120 min.

Neurological and cognitive outcomes of patients were assessed at 1 month using the Global Deterioration Score (GDS) with a score range of 1–7 [55]. Outcome was partitioned between those with complete recovery (GDS = 1) or not (all GDSs > 1). Blood samples obtained during the initial ED visit were collected in Cyto-chex BCT tubes (Streck, Inc., Omaha, NE, USA), numerically coded, and analyzed between 3 and 10 days later. Samples from South Korea were shipped via express mail to the senior author’s lab. Prior work has shown that MPs and neutrophil characteristics remain unchanged when samples are processed within 3 weeks from the time of acquisition and refrigeration is not required.

### 4.3. Animals

Male and female C57BL/6J mice (8–12 weeks old; Mus musculus) were purchased from the VR Breeding facility at the University of Maryland, Baltimore, and housed in a university-managed vivarium with free access to standard rodent chow (Laboratory Rodent Diet 5001; PMI Nutrition Inc., Brentwood, MO, USA) and water ad libitum.

Mice of both sexes, used in approximately equal numbers, were randomly assigned to breathe room air (control) or undergo a standardized CO exposure protocol consisting of 1000 ppm CO for 40 min followed by 3000 ppm for 20 min. No mortality occurred, and no animals exhibited overt signs of physiological distress during or after exposure. Where indicated, mice received intraperitoneal injections of anakinra (100 mg/kg), a polyclonal anti-mouse IL-1β neutralizing antibody (BioXcell, Cat. No. BE0246; 50 µg/mouse), or a nonspecific isotype control antibody (BioXcell, Cat. No. BE0089; 50 µg/mouse) administered either prior to or following CO exposure.

Animals were assigned to treatment groups using block randomization and euthanized at designated time points ranging from 2 h to 21 days post-exposure for blood and tissue collection. Mice were anesthetized with intraperitoneal ketamine (100 mg/kg) and xylazine (10 mg/kg), and blood was collected via aortic puncture using heparinized syringes.

All analyses were performed by investigators blinded to group allocation to ensure unbiased interpretation of results. No animals were excluded from analysis. To maximize information gained while minimizing animal use, both blood and tissue samples were collected from each animal whenever feasible.

### 4.4. Microparticles Preparation and Analysis

Microparticles were isolated from human and mouse blood, as well as from deep cervical lymph nodes, following previously published protocols [11,12,14]. In brief, blood MPs were prepared by centrifuging heparinized blood at 1500× *g* for 5 min, adding EDTA to the supernatant to achieve a final concentration of 12.5 mM to prevent MP aggregation, and then centrifuging at 15,000× *g* at 4 °C for 30 min. MPs from DCLN were prepared by homogenizing DCLN in digestion buffer (DMEM, 2% FBS containing 250 μg dispase) and filtered through a 40 μm cell strainer. To prevent particle adhesion, 0.1 mL of 50 mM EDTA/mL of node suspension was added, and the mixture centrifuged at 600× *g* for 5 min to remove large particles. The supernatant was further centrifuged at 15,000× *g*, 4 °C for 30 min to collect MPs. MPs in the supernatant were stained and analyzed by flow cytometry. For surface staining in flow cytometric analysis, 10 µL of MPs were incubated for 30 min at room temperature in the dark with optimized concentrations of fluorescently labeled reagents in 100 µL of filtered Annexin V binding buffer.

Intraparticle IL-1β expression was assessed by flow cytometry after permeabilizing MPs with the FIX & PERM™ Cell Permeabilization Kit (Invitrogen, Cat# GAS003) and staining intracellularly with a fluorochrome-conjugated anti-IL-1β antibody, according to the manufacturer’s instructions.

The same protocol was followed for all analyses. Forward and side scatter were recorded on logarithmic gain, and photomultiplier tube voltages and trigger thresholds were optimized specifically for the detection of submicron particles and exclusion of debris. Calibration beads of 0.3 µm, 1.0 µm, and 3.0 µm were analyzed at the start of each experiment to standardize particle sizing and gating parameters. MPs were defined as Annexin V-positive events falling within the 0.3–1.0 µm size gate. This strict gating strategy removes fragments and extracellular vesicles and allows capture of only MPs. All the markers used in this study were gated over the Annexin V-positive events falling within the 0.3–1.0 µm size gate. MP concentrations were quantified based on the exact volume acquired, as calculated by MACSQuantify™ software (version 2.13.3).

All reagents and solutions were rendered sterile and passed through a 0.1-µm filter (EMD Millipore, Billerica, MA, USA) before use. MPs characterization was performed using an 8-color, triple-laser MACSQuant^®^ Analyzer (Miltenyi Biotec, Auburn, CA, USA). Calibration of instrument and setting up gating strategy by using sizing beads of 0.3 µm, 1.0 µm, and 3.0 µm were performed according to previously published methods [45]. MP concentrations were quantified based on the exact volume acquired, as calculated by MACSQuantify™ software (version 2.13.3).

### 4.5. Purification of Phalloidin-Positive MPs

Separation of MPs expressing filamentous (F)-actin on their surface was performed using magnetic bead-based isolation following a previously published protocol [41]. In summary, MP suspensions were labeled with biotin-conjugated phalloidin followed by streptavidin-conjugated magnetic microbeads. F-actin-positive (F+) MPs were isolated using a µMACS magnetic separation system, in which F+ MPs were retained within the magnetic column while F-actin-negative (F−) MPs passed through. The F+ MPs fraction was subsequently eluted according to the manufacturer’s instructions.

### 4.6. Quantification of IL-1β in Microparticles

The concentration of IL-1β associated with human and murine total MPs, F+ MPs, and F− MPs was measured using commercially available ELISA kits specific to each species, following the manufacturers’ recommended procedures. MPs were isolated as described in our flow cytometry preparation workflow [11,12,14], then subjected to high-speed ultracentrifugation at 100,000× *g* for 60 min to obtain an MP-enriched pellet. The pellet was resuspended and lysed in lysis buffer containing (20 mM Tris, 150 mM NaCl, 1% Nonidet P-40, 0.5% sodium deoxycholate,1 mM EDTA and 0.1% SDS (pH 7.5) with protease inhibitors cocktail (Sigma, Singapore)) to preserve cytokine integrity. Total protein concentration in the MP lysates was quantified, normalized to 5 mg/mL to ensure sample comparability, and an aliquot containing 20 µg of total protein was used for ELISA and Western blot. This approach allowed assessment of IL-1β specifically packaged within MPs, providing a standardized basis for comparing IL-1β cargo content across experimental groups. Prior work has failed to find IL-1β in supernatants, but these findings were not with CO patients [51]. Therefore, studies were conducted with 18 separate CO patient supernatants following our published protocol and no IL-1β was detected (detection limit 1.2 pg).

### 4.7. Neutrophil Activation

Whole fixed blood from humans and mice was processed for neutrophil (PMN) activation analysis as previously described [11,12,14]. Briefly, 50 µL of whole blood was stained with optimized fluorochrome-conjugated antibodies, diluted in PBS, and analyzed by flow cytometry. Neutrophils were identified using Ly6G (mouse) or CD66b (human) as the trigger parameter, and activation was assessed by measuring CD18 and myeloperoxidase (MPO) expression.

### 4.8. Lymphocyte Proliferation

Peripheral lymph nodes from mice were mechanically dissociated by gently pressing the tissue through a sterile wire mesh screen using the plunger of a glass syringe in ice-cold complete RPMI medium (RPMI 1640 supplemented with 10% FBS and 50 µg/mL gentamicin). The resulting cell suspension was aspirated several times through a 25-gauge needle to disperse residual clumps, washed once with complete RPMI, and centrifuged at 433× *g* for 5 min at 4 °C. The supernatant was discarded, and the cell pellet was resuspended in 1 mL of complete RPMI medium. After counting, cells were plated at 3 × 10^5^ cells per well in 96-well plates.

Cells were stimulated with 5 µg/well myelin basic protein (MBP) and incubated at 37 °C in a humidified CO_2_ incubator for 48 h. Following incubation, wells were washed with ice-cold PBS, and 0.5 µCi of [^3^H]-thymidine (1 Ci = 37 GBq) was added to each well. Plates were incubated for an additional 21 h to allow incorporation of the radiolabel.

Cells were then washed and harvested onto glass fiber filters using a cell harvester. Filters were air dried at room temperature and either punched into scintillation vials or processed directly for quantification using a beta plate counter. Scintillation fluid was added according to the manufacturer’s instructions. Radioactivity was measured as counts per minute (cpm) using a beta scintillation counter under laboratory-standardized settings.

### 4.9. Assessment of Vascular Injury

Vascular injury was quantified by measuring the uptake of perfused lysine-fixable tetramethyl rhodamine-conjugated dextran (2 × 10^6^ Da; Invitrogen, Carlsbad, CA, USA) in brain and skeletal muscle homogenates enriched for endothelial content using a colloidal silica extraction method [41,42]. Rhodamine fluorescence was measured to assess vascular leakage, and all values were normalized to a control mouse included in each experiment to account for batch-to-batch variability in dextran preparation.

### 4.10. CO-Induced Neuroinflammation

Endothelium-enriched brain protein fractions were prepared according to previously described procedures [41,42]. Total protein concentration was measured using the BCA protein assay, and samples were stored at −80 °C. Proteins (20 μg) were separated on 4–12% precast gels and transferred onto nitrocellulose membranes using the iBlot™ 3 system (Thermo-Fischer, Waltham, MA, USA). Membranes were blocked with Intercept^®^ (PBS) Blocking Buffer (Li-Cor) for 1 h at room temperature followed by overnight incubation at 4 °C with primary antibodies targeting AQP4, MPO, CD36, TSP1, Ly6G, NF-κB, pNF-κB, Phosphatidyl serine, IL-1β and β-actin. The membranes were then rinsed with PBST (three washings for 10 min), incubated with IRDye-labeled secondary antibodies for 1 h at room temperature, rinsed again, and visualized using an Odyssey infrared imaging system (Li-Cor, Lincoln, NE, USA).

### 4.11. Cognitive Deficits Following CO Exposure

Cognitive deficit in mice was measured using attentional set shifting and reversal learning procedures adapted from previously published methodology [41,46]. Beginning three days after CO exposure, mice were acclimated to retrieve a food reward by digging in small bowls and testing was completed 3 weeks after CO exposure. Once reliable digging behavior was established, animals completed a series of discrimination stages designed to assess learning, rule acquisition, and cognitive flexibility.

The sequence began with a simple discrimination (SD) in which the bowls differed only in odor cues. After meeting the criterion of eight consecutive correct responses, mice advanced to a compound discrimination (CD) that introduced an additional dimension (digging medium) while keeping odor as the relevant cue. A subsequent intradimensional shift (IDS) required mice to apply the same rule using newly presented stimuli.

After achieving criterion at this stage, mice underwent a reversal learning (R) phase in which reward contingencies associated with the odor cues were switched. Animals then completed a second intradimensional shift (IDS2) followed by an extradimensional shift (EDS), during which the predictive stimulus dimension shifted from odor to digging medium. Increased trials needed to complete the EDS relative to IDS2 indicated the formation of an attentional set.

Testing proceeded over three to five consecutive days, depending on individual performance, allowing assessment of rule learning, flexibility, and shifting of attentional strategies. Demonstration of an attentional set formation was defined as requiring at least two more trials to solve the EDS relative to the preceding IDS (IDS2).

### 4.12. Statistical Analysis

Results are presented as mean ± standard deviation (SD) from at least four independent experiments, analyzed using SigmaStat (Jandel Scientific, San Jose, CA, USA). Data normality was evaluated with the Shapiro–Wilk test. Details of the statistical methods used for each assay are provided in the corresponding figure legends. For two-group comparisons, two-tailed, unpaired Student’s *t*-test was used when data were normally distributed. For comparisons among multiple groups in MPs, inflammation and vascular injury assays, Kruskal–Wallis test (repeated measures ANOVA on Ranks) was used, followed by Dunn’s post hoc test with Bonferroni adjustment for pairwise comparisons. Behavioral outcomes with significant overall group differences were further analyzed using Tukey’s post hoc tests following one-way. A *p*-value < 0.05 was considered indicative of statistical significance throughout all analyses.

## 5. Conclusions

In summary, this study identifies F^+^MPs as a carrier of IL-1β, which plays a central mediator linking carbon monoxide exposure to systemic inflammation, neurovascular injury, and long-term cognitive dysfunction. By demonstrating that blockade of IL-1β signaling suppresses MPs generation, neutrophil activation, vascular permeability, neuroinflammation, and executive function deficits, our findings redefine IL-1β-loaded F^+^MPs as actionable therapeutic targets rather than passive biomarkers of injury. These results provide strong mechanistic rationale for repurposing IL-1-targeted therapies, such as anakinra, as adjunctive treatments in CO poisoning and suggest broader relevance for MP-mediated inflammatory pathways in other forms of neurotoxic and neuroinflammatory disease.

## Figures and Tables

**Table 1:** Clinical results for CO-poisoned patients who had good outcome (GDS = 1) versus those who sustained NS by 1 month (GDS > 1); *n* = 27 each group.

Characteristic	GDS = 1	GDS > 1
Age (years)	41.4 ± 14.8	43.9 ± 19.5
# male (%)	14 (58%)	11 (47%)
COHb%	16.5 ± 12.8	16.4 ± 10.5
Pg IL-1β/million MPs	33.0 ± 32.0	43.8 ± 42.0
Lactate (mmol/L)	3.8 ± 7.1	3.1 ± 2.3
Troponin (ng/mL)	423 ± 1022	3864 ± 5808 *
# LOC (%)	10 (42%)	11 (46%)
Intubated (%)	5 (19%)	9 (38%)
Acute GCS	13.1 ± 3.0	12.8 ± 4.0
GDS at 1 month	1.0 ± 0.0	4.1 ± 1.9

The rows show age, sex, %COHb level, biomarkers from blood obtained in the ED including IL-1β (pg)/ 1 million MPs, blood lactate and troponin, number (and %) who sustained a loss of consciousness (LOC), were intubated in the ED, and first Glasgow Coma Score (GCS) score on ED arrival, and the Global Deterioration Score (GDS) for the 1-month follow-up (good outcome, GDS = 1, those with neurological sequelae, GDS > 1) [40]. Values are mean, ± SD for continuous variables, and count (proportion) for categorical variables. * *p* < 0.01, there are no other statistically significant differences between the groups.

**Table 2:** Flow cytometric characterization of mouse blood MP subgroups.

Groups	% Ly6G	%F-Actin	%CD41	%CD31/CD41	%GFAP	% MBP	%NPR	%TSP1	%TMEM	%CD36
Control (*n* = 12)	2.4 ± 1.0	3.5 ± 1.3	14.8 ± 2.7	0.9 ± 0.5	33.8 ± 1.3	12.7 ± 1.3	23.1 ± 2.1	3.9 ± 1.2	19.1 ± 2.2	17.7 ± 3.6
CO-2hrs (*n* = 7)	6.2 ± 1.0 *	6.9 ± 1.2 *	21.6 ± 4.6 *	2.9 ± 0.8 *	37.4 ± 1.8	16.0 ± 0.7 *	28.4 ± 2.0	5.5 ± 0.7	25.5 ± 2.3	23.0 ± 4.12
Anakinra control (*n* = 6)	2.4 ± 0.5	4.0 ± 0.9	22.2 ± 3.6	1.7 ± 0.6	33.2 ± 1.7	13.3 ± 0.6	29.1 ± 1.5	2.8 ± 0.3	25.3 ± 1.6	18.2 ± 2.12
Anakinra pre-CO (*n* = 4)	2.0 ± 0.9	2.4 ± 0.3	24.0 ± 1.1	1.6 ± 0.3	34.4 ± 0.1	14.0 ± 0.3	31.4 ± 1.4	3.5 ± 0.2	24.8 ± 1.9	21.0 ± 2.4
Anakinra post-CO (*n* = 4)	1.7 ± 0.3	3.9 ± 0.5	24.7 ± 0.2	1.9 ± 0.2	33.5 ± 0.6	14.0 ± 0.5	28.2 ± 0.7	3.2 ± 0.4	24.4 ± 0.6	19.7 ± 1.7
Anakinra 3 week post-CO (*n* = 12)	2.5 ± 0.6	3.8 ± 1.1	16.3 ± 3.9	0.9 ± 0.7	23.0 ± 0.8	9.7 ± 0.9	29.3 ± 0.6	2.8 ± 0.4	21.9 ± 0.4	14.3 ± 1.10
IL-1β post CO (*n* = 6)	2.3 ± 1.0	3.8 ± 0.7	22.7 ± 2.9	1.8 ± 0.2	30.9 ± 2.5	12.5 ± 0.2	26.4 ± 0.3	2.1 ± 0.1	21.9 ± 0.5	15.5 ± 1.10

Blood MPs were probed for proteins specific to the CNS such as from astrocytes (glial fibrillary acidic protein, GFAP), microglia (transmembrane protein119, TMEM), oligodendroglia (myelin basic protein, MBP) and neurons (neuronal pentraxin receptor, NPR), neutrophils (Ly6g), platelets (CD41a), CD36, and those expressing TSP-1 and F-actin that were assessed by fluorescent labeled phalloidin binding. Experimental groups include: untreated controls; mice exposed to CO and euthanized 2 h later; control mice injected intraperitoneally with sterile anakinra (100 mg/kg) and euthanized 2 h later; mice administered anakinra (100 mg/kg IP) either before or immediately after CO exposure and euthanized at 2 h or 3 weeks later; and mice administered anti-IL-1β antibody (50 μg/mice) immediately after the CO exposure and euthanized 2 h later. The data represents mean ± SD; *n* for each group shown on left column; (*) indicates *p* < 0.05 vs. control (Kruskal–Wallis test).

**Table 3:** Flow cytometric characterization of deep cervical lymph node MP subgroups.

Groups	% GFAP	%MBP	%NPR	%TSP	%TMEM
Control (*n* = 12)	36.9 ± 6.5	17.1 ± 1.9	25.2 ± 5.2	4.4 ± 1.6	18.2 ± 6.7
CO-2hrs (*n* = 7)	51.6 ± 5.5 *	25.4 ± 2.9 *	35.6 ± 4.2 *	10.5 ± 3.1 *	40.5 ± 11.2 *
Anakinra control (*n* = 6)	37.6 ± 7.2	16.7 ± 2.1	16.7 ± 2.1	2.4 ± 1.3	26.2 ± 6.1
Anakinra pre-CO (*n* = 4)	38.1 ± 2.2	18.4 ± 0.6	16.7 ± 0.6	2.2 ± 0.8	22.6 ± 1.2
Anakinra post-CO (*n* = 4)	41.9 ± 3.1	13.7 ± 1.2	16.7 ± 1.2	2.1 ± 0.9	17.2 ± 1.9
Anakinra post-CO-3 week (*n* = 12)	29.2 ± 3.8	15.4 ± 2.4	16.7 ± 2.4	2.8 ± 1.1	32.5 ± 2.4
IL-1β post-CO (*n* = 6)	40.8 ± 6.1	19.6 ± 2.9	16.7 ± 2.9	4.3 ± 1.7	32.1 ± 6.9

Shown are the percentages of MPs positive for cell-specific markers, including astrocytes (GFAP), oligodendroglia (MBP), neurons (NPR), microglia (TMEM119), and MPs expressing TSP-1. Experimental groups are described in Table 2. The data represents mean ± SD; *n* for each group shown on left column; (*) indicates *p* < 0.01 vs. control (Kruskal–Wallis test).

## Data Availability

All data needed to evaluate the conclusions in the paper are present in the paper and will be made available to responsible parties on request.

## Supplementary Materials

The following supporting information can be downloaded at: https://www.mdpi.com/article/10.3390/ijms27177880/s1.

## Abbreviations

The following abbreviations are used in this manuscript:

ASSTR	Attentional Set Shifting and Reversal Task
BBB	blood–brain barrier
CNS	central nervous system
DCLN	deep cervical lymph nodes
ED	Emergency Department
ELISA	Enzyme-linked immunosorbent assay
F-actin	filamentous-actin
GDS	Global Deterioration Score
MBP	myelin basic protein
MPs	microparticles
NLRP3	nucleotide-binding oligomerization domain-like receptor, pyrin domain containing 3
NS	neurological sequelae
