# Supplementary material for: Microparticles Expressing IL-1β Are Linked to Neuroinflammation and Cognitive Decline After Carbon Monoxide Poisoning
Source: Int J Mol Sci. 2026 Sep 3;27(17):7880. doi: 10.3390/ijms27177880 (PMC13566203; doi:10.3390/ijms27177880)
Supplement: Supplementary file 1 [file ijms-27-07880-s001.zip › ijms-4470583-supplementary.pdf]

Supplemental Table S1: Clinical findings for all patients with CO poisoning separated between those with GDS=1 at 1 month versus those with GDS >1 (two outer columns), and the two inner columns showing those where IL-1β was measured. Rows demonstrate age, % male, %COHb, blood lactate, and troponin when first seen in the ED, % of those who sustained a loss of consciousness (LOC) and who were intubated in the ED, first Glasgow Coma Score (GCS) on ED arrival, and GDS score at 1 month follow- up. Values are mean ± SD for continuous variables and count (proportion) for categorical variables. Statistical comparison was made between good outcome and neurological sequelae groups with P <0.05 indicated by *. There were no significant differences between the total GDS =1 group and the group where IL-1β was measured, nor between the total GDS >1 group and the group where IL-1β was measured.

	All GDS=1 (77)	GDS=1 (27)	GDS>1 (27)	All GDS>1 (37)
Age	41.4±14.8	43.7±14.9	43.9±19.5	47.3±19.1
# male (%)	64%	48%	47%	41%
COHb%	16.5±12.8	16.2±12.7	16.4±10.5	19.4±11.5
Lactate	3.8±7.1	3.8±9.2	3.1±2.3	3.8±3.3
Troponin	423±1021	517±1099	3864±5808*	2429±4638*
LOC	42%	38%	46%	46%
Intubated	19%	14%	38%*	46%*
Acute GCS	13.1±3.0	13.4±2.9	12.8±4.0	11.3±4.7
GDS at 1 mo	1.0±0.0	1.0±0.0	4.1±1.9*	3.7±1.9*

Supplemental Table S2. Comparison of GDS scores between current group of 27 and total group (n=37):

GDS value	This paper (27)	Total (37)
2	9 (33%)	16 (43%)
3	3 (11%)	4 (11%)
4	5 (19%)	7 (19%)
5	3 (11%)	3 (8%)
7	7 (26%)	7 (19%)

Supplemental Table S2: Data show the distribution of GDS scores for the 27 with poor outcome for whom IL-1β was measured and the larger group (n=37) in our earlier report.
